# An explorative analysis of *ERCC1-*19q13 copy number aberrations in a chemonaive stage III colorectal cancer cohort

**DOI:** 10.1186/1471-2407-13-489

**Published:** 2013-10-21

**Authors:** David Hersi Smith, Ib Jarle Christensen, Niels Frank Jensen, Bo Markussen, Sven Müller, Hans Jørgen Nielsen, Nils Brünner, Kirsten Vang Nielsen

**Affiliations:** 1 R&D, Dako A/S, Produktionsvej 42, Glostrup DK-2600, Denmark; 2 Section for Molecular Disease Biology, Department of Veterinary Disease Biology, Faculty of Health and Medical Sciences, University of Copenhagen, Dyrlægevej 88, Frederiksberg DK-1870, Denmark; 3 Finsen Laboratory, Rigshospitalet and Biotech Research and Innovation Centre (BRIC), University of Copenhagen, Copenhagen Biocenter, Ole Maaloevs Vej 5, building 3, 3rd floor, Copenhagen N DK-2200, Denmark; 4 Laboratory of Applied Statistics, Department of Mathematical Sciences, Faculty of Science, University of Copenhagen, Universitetsparken 5, Copenhagen Ø DK-2100, Denmark; 5 Department of Surgical Gastroenterology 360, Hvidovre Hospital, Kettegård Allé 30, Hvidovre DK-2650, Denmark; 6 Institute of Clinical Medicine, Faculty of Health and Medical Sciences, University of Copenhagen, Blegdamsvej 3B, Copenhagen N DK-2200, Denmark; 7 Current address: Centre for Innovation and Research, Ole Maaloevs Vej 3, Copenhagen N DK-2200, Denmark

**Keywords:** ERCC1, ERCC4, XPF, Colorectal cancer, FISH, Prognosis

## Abstract

**Background:**

Platinum-based chemotherapy has long been used in the treatment of a variety of cancers and functions by inducing DNA damage. ERCC1 and ERCC4 are involved in the removal of this damage and have previously been implicated in resistance to platinum compounds. The aim of the current investigation is to determine the presence, frequency and prognostic impact of *ERCC1* or *ERCC4* gene copy number alterations in colorectal cancer (CRC).

**Methods:**

Fluorescent in situ hybridization probes directed at *ERCC1* and *ERCC4* with relevant reference probes were constructed. Probes were tested in a CRC cell line panel and in tumor sections from 152 stage III CRC chemonaive patients. Relationships between biomarker status and clinical endpoints (overall survival, time to recurrence, and local recurrence in rectal cancer) were analyzed by survival statistics.

**Results:**

*ERCC1-*19q13 copy number alterations were observed in a single cell line metaphase (HT29). In patient material, *ERCC1-*19q13 copy number gains (*ERCC1-*19q13/CEN-2 ≥ 1.5) were detected in 27.0% of specimens, whereas *ERCC1*-19q13 deletions (*ERCC1-*19q13/CEN-2 < 0.8) were only detected in 1.3%. *ERCC1*-19q13 gain was significantly associated with longer survival (multivariate analysis, HR: 0.45, 95% CI: 0.20-1.00, p = 0.049) in patients with colon tumors, but not rectal tumors. No *ERCC4* aberrations were detected and scoring was discontinued after 50 patients.

**Conclusions:**

*ERCC1-*19q13 copy number gains occur frequently in stage III CRC and influences survival in patients with colon tumors. Future studies will investigate the effect of *ERCC1*-19q13 aberrations in a platinum-treated patient population with the aim of developing a predictive biomarker profile for oxaliplatin sensitivity in CRC.

## Background

Oxaliplatin, in combination with 5-Fluorouracil (5-FU, capecitabine) and additional biological agents, presents one of two chemotherapy options available for the treatment of advanced colorectal cancer (CRC). Similar to other platinum compounds, oxaliplatin exerts its cytotoxic activity by causing damage to cellular DNA in the form of helix distorting DNA-platinum adducts, such as intra- and interstrand DNA and DNA-protein crosslinks [1,2]. Several DNA repair systems are involved in the removal of this damage, including nucleotide excision repair (NER) [3]. NER is initiated by recruitment of several proteins to the site of damage. ERCC1 and ERCC4 (also known as XPF) form a heterodimer with endonuclease activity, which is recruited at 5′ to the DNA lesion. Following incision by the complex, another endonuclease (ERCC5, also known as XPG) cleaves at 3′ to the lesion, allowing removal of the damage nucleotide(s). The missing fragment is replaced and ligated [4]. Due to a central role in NER, as well as interstand crosslink repair [5], the ERCC1-ERCC4 heterodimer has been widely studied in relation to platinum resistance.

In a landmark study, Shirota and colleagues found a link between low ERCC1 mRNA expression levels and longer survival in a stage IV CRC oxaliplatin-treated patient cohort [6]. Numerous other studies have attempted to link ERCC1 protein levels to platinum sensitivity [7-9], however these studies have relied on the use of a particular monoclonal antibody, which has recently been found to bind an unrelated protein, raising questions towards the validity of these results [10,11]. Interestingly, studies of gene copy number alterations involving the *ERCC1* locus at 19q13 and *ERCC4* locus at 16p13.12 are very limited in number and have not been performed in CRC [12-14].

Predictive biomarkers can encompass both prognostic and predictive components, a phenomenon which may hamper the detection of a beneficial effect from treatment, unless the prognostic element has been investigated and mapped [15]. With the aim of identifying a predictive biomarker profile for oxaliplatin sensitivity, we have constructed two novel FISH probes directed at *ERCC1* and *ERCC4* genes, as well as relevant reference probes. These were subsequently tested these in a CRC cell line metaphase panel to identify potential aberrations. Both probe combinations were subsequently tested in a chemonaive stage III CRC patient cohort to determine the presence, frequency and prognostic impact of *ERCC1*/*ERCC4* gene aberrations. Based on the collected FISH data, scoring guidelines were established for future use.

## Methods

### Patients

A total of 154 patients with histologically proven stage III adenocarcinomas were selected as previously described [16,17]. All tumors were resected and patients did not receive adjuvant chemo- and/or radiotherapy as this was not part of the standard CRC treatment in Denmark at the time (1991–1993). Patients were enrolled in the RANX05 clinical trial and were randomized to receive Ranitidine or placebo for up to five years. The aim of the RANX05 study was to evaluate whether Ranitidine, a histamine type 2 receptor antagonist, could provide a survival benefit when used as single agent in the adjuvant setting. Ranitidine had no effect on survival [18]. The RANX05 trial was conducted in accordance with the Helsinki II Declaration and was approved by the Danish National Board of Health (2760-419-1989), Data Protection Agency (1991-1110-751) and Central National Ethics Committee (KF 01-2045/91). The approval included collection of tissue specimens for subsequent analysis of biological markers (KF 01-078/93).

### Metaphase preparation

Preparation of CRC cell lines metaphases has been reported previously [16]. Briefly, Colo-205, HCC-2998, HCT-15, HCT-116, HT29, KM12, and SW620 were obtained from the NCI/Development Therapeutics Program, while DLD-1, LoVo, and LS-174 T were obtained from the American Tissue Culture Collection. Cell lines were maintained at 37°C, 5% CO_2_ in relevant growth medium. Once cultures reached _~_ 70% confluence, colcemid (Invitrogen, Carlsbad, USA) was added to the culture. After 2 h, cells were harvested and a hypotonic treatment was carried out. Cells were fixed and dripped onto glass slides.

### FISH

#### Probe design

The *ERCC1* gene probe, consisting of bacterial artificial chromosome (BAC) clones RP11-752G9 (Invitrogen) and CH17-274E15 (BACPAC Resources, Children’s Hospital Oakland Research Insitute, Oakland, USA), covers an approximately 377 kB region containing *ERCC1*, as well as several other genes: *MARK4, CKM, KLC3, ERCC2, PPP1R13L, CD3EAP, FOSB, RTN2, PPM1N, VASP, OPA3, GPR4* and partially *EML2*. Due to the additional coverage of genes other than *ERCC1*, this FISH probe will be referred to as *ERCC1*-19q13. Following DNA purification from culture, BAC clones were labeled with the Texas Red fluorochrome by nick translation. This gene probe was combined with a centromere 2-specific probe (Dako, Glostrup, Denmark), consisting of several FITC-labeled peptide nucleic acid (PNA) oligomers, which has previously been found to reflect cellular ploidy levels (described in [16]). Due to the nature of the repetitive elements in the centromeric region of chromosome 19, it was not possible to generate a centromere 19-specific probe. The *ERCC4* gene probe, consisting of BACs CTD-3160 N7 (Invitrogen) and RP11-99H5 (Invitrogen), covers an approximately 348 kb region, which only contains the *ERCC4* gene and was prepared as described above. This gene probe was combined with a centromere 16-specific probe (CEN-16) (Dako). Both gene probes were mixed their relevant reference probes in the IQFISH Buffer (Dako) [19].

#### FISH procedure

The applied FISH reagents were from the Cytology FISH Accessory Kit (K5499) and the Histology FISH Accessory Kit (K5799) (Dako). The FISH procedure has previously been described [16]. Briefly, metaphase specimens were fixed and dehydrated. Once dry, FISH probe was dripped onto slide and the specimens was denatured and hybridization was performed. Excess probe was removed by incubation in stringency buffer and slides were subsequently washed, dehydrated, dried and mounted. Hybridization to FFPE specimens (thickness: 3 μm) was performed according to the manufacturer’s recommendations (Dako). Briefly, slides were prepared by heat pretreatment and pepsin digestion. Slides were subsequently treated as described above.

#### FISH scoring

The applied FISH scoring methods have previously been described [16]. FISH signals were scored according to *TOP2A* FISH pharmD×™ guidelines (Code K5333, package insert, 1st edition, 2008.01.18) at 1000× magnification in the Texas Red/FITC double filter provided the signals were, as a minimum, visible at 200× magnification in the appropriate filter. Although 60 nuclei were scored for each sample, only nuclei harboring both gene- and reference signals were included for further analysis.

To determine the presence and mechanism of *ERCC1*-19q13 and *ERCC4* copy number aberrations in cell lines, signal locations and numbers were noted for 50 metaphases for each cell line. The total number of chromosomes for each cell line has previously been determined [16].

### Statistical methods

SAS 9.2 (SAS Institute, Cary, USA) was used to perform all descriptive and survival statistical analyses. R version 2.15.2 was used in scoring method optimization.

#### Scoring method optimization

Gene to centromere ratios were calculated by including the first 10, 20 or 30 nuclei, determining *ERCC1*-19q13 status and comparing this to the status after inclusion of all relevant nuclei. Concordance was calculated by use of Kendall’s tau [tau = (agree-disagree)/(agree + disagree)]. A borderline interval near the cut-off value (1.5), where additional nuclei must be included (for 10 nuclei, an additional 10 nuclei have to be scored; for 20 nuclei, an additional 20 nuclei have to be scored and so forth) was defined as greater than or equal to 1.35 (min) and less than 1.65 (max). Concordance and mean number of nuclei scored were calculated with and without borderline intervals.

#### Survival analysis

For the survival analysis three clinical endpoints were considered: overall survival (OS, time to death by any cause), time to recurrence (TTR, time to any event related to colorectal cancer) and time to local recurrence in rectal cancer (LR) (described in detail in [17]). Kaplan-Meier estimates of survival probabilities are presented for the dichotomized variables. Multivariate Cox regression was done adjusting for gender, age (per 10 year difference in age) and tumor localization (colon and rectum). Cox regression analysis was applied for the analyses. Models were validated by assessing the proportionality assumption and linearity for continuous covariates employing Schönfeld and martingale residuals. *ERCC1*-19q13 and CEN-2 copy numbers, as well as the *ERCC1*-19q13/CEN-2 ratio, were log transformed (base 2) when analyzed as a continuous variable and therefore the hazard ratio (HR) reflected a two-fold difference for these variables. Results are presented by hazard ratios hazard ratios with 95% confidence intervals (CI) and p-values. P-values were two-sided and considered significant at 0.05 for all main effects.

## Results

### *ERCC1-*19q13/*ERCC4* FISH in cell line panel

**Figure 1:**
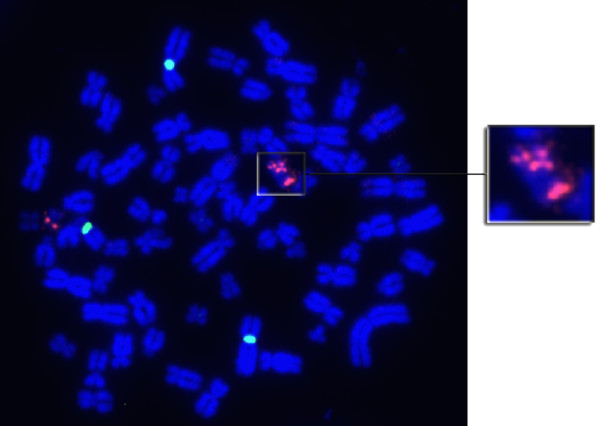
**Metaphase of HT29.** HT29 is a near-triploid CRC cell line, which was found to harbor a 19q isochromosome fused with an additional (at least partial) duplicated 19q chromosome fragment.

To determine the presence and mechanism of *ERCC1*-19q13/*ERCC4* gene copy number alterations, metaphases were prepared from a panel of ten CRC cell lines. Metaphase preparation was successful for all but one cell line (LS174T). Hybridization with the *ERCC1*-19q13/CEN-2 probe revealed the presence of a gene copy number aberration in HT29. As shown in Figure 1, HT29 appears to harbor a copy of chromosome 19 with a single *ERCC1*-19q13 signal, as well as one harboring 3 signals, producing an overall *ERCC1*-19q13/CEN-2 ratio of 1.33. No aberrations were detected with *ERCC4*/CEN-16.

### Stage III CRC patient material

#### Samples

**Figure 2:**
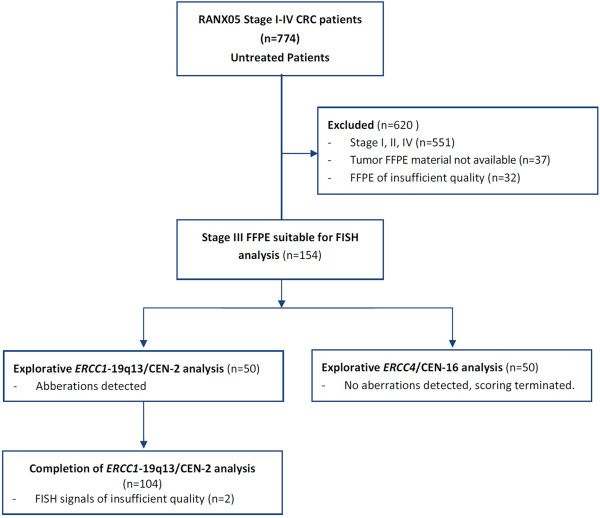
CONSORT flow diagram describing the selection method of samples included in this study.

**Figure 3:**
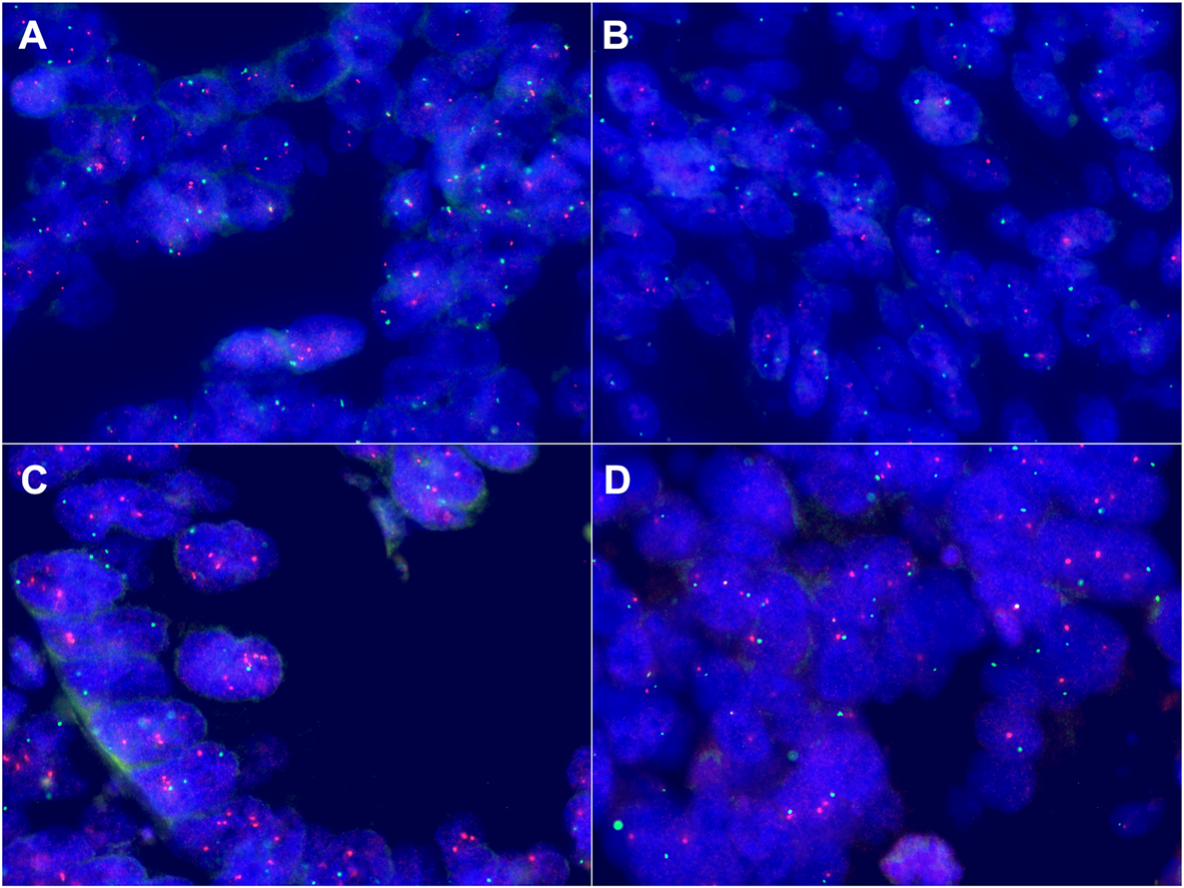
**Representative images of FISH analysis in stage III CRC specimens - taken at 1000x magnification. A**: *ERCC1*-19q13 Normal (ratio 0.99). **B**: *ERCC1*-19q13 Deletion (ratio 0.65). **C**: *ERCC1*-19q13 Gain (ratio 1.90). **D**: *ERCC4* (ratio 1.21).

A total of 154 FFPE tumor blocks were available for FISH. As shown in Figure 2, fifty randomly selected samples were initially assessed with both *ERCC1*-19q13/CEN-2 and *ERCC4*/CEN-16 FISH to determine the presence of gene aberrations. No aberrations were detected with *ERCC4*/CEN-16 and hybridization was discontinued. Following the detection of aberrations, *ERCC1-*19q13/CEN-2 hybridization to FFPE patient specimens was continued and was successful for 152 (98.7%) of a total of 154 available samples, specifically 81 colon and 71 rectum specimens. Examples of FISH analysis can be viewed in Figure 3. Baseline characteristics including age, gender, tumor location, number of local recurrences and distant metastases have been described elsewhere [17].

#### *ERCC1*-19q13 FISH

To improve upon assay sensitivity to detect *ERCC1*-19q13 copy number alterations, only nuclei harboring both *ERCC1*-19q13 and CEN-2 signals were included for subsequent analysis, which resulted in a median of 53 nuclei scored for each tumor specimen (range: 42–60). In the patient material, average *ERCC1*-19q13 counts ranged from 1.08 to 4.37 signals per nucleus with a median of 2.11, whereas CEN-2 ranged from 1.29 to 2.32 with a median of 1.60. Tumor heterogeneity was not observed.

The *ERCC1*-19q13/CEN-2 ratio ranged from 0.65 to 2.47 with a median of 1.28. As shown in Table 1, when applying relevant ratio cut-off values, tumor specimens could be separated into three different *ERCC1*-19q13 statuses. The use of a ratio cut-off of 1.5, reflecting an additional gene copy in a diploid cell, classified 41 (27.0%) specimens as harboring an *ERCC1*-19q13 gain. *ERCC1*-19q13 gain was more frequently observed in rectal tumors (24/71 – 33.8%) than colon tumors (17/81 – 21.0%). To identify specimens with gene loss (deletion), a ratio of 0.8 was used as a cut-off, which yielded two samples in this category, both of which were colon tumors (2/81). The remaining samples were classified as ‘*ERCC1*-19q13 Normal’.

**Table 1:** ***ERCC1*-19q13 status in 152 CRC samples with applied cut-offs, observed ratio ranges for each *ERCC1*-19q13 status, and the number of specimens in each of the status groups**

***ERCC1*-19q13 Status**	**Cut-off values**	**Ratio range**	**Frequency n (%**^**a**^**)**
**Deletion**	< 0.8	0.65-0.69	2 (1.3)
**Normal**	≥ 0.8 and < 1.5	0.97-1.48	109 (71.7)
**Gain**	≥ 1.5	1.52-2.47	41 (27.0)

^a^% denotes percentage of samples relative to total number of samples.

#### *ERCC4* FISH

In the initial 50 FFPE specimens, the *ERCC4*/CEN-16 ratio ranged from 0.90 to 1.39 with a median of 1.09. Mean *ERCC4* signals per nucleus ranged from 1.37 to 2.35 (median = 1.82), whereas CEN-16 ranged from 1.40 to 2.06 with a median of 1.68. Due to a lack of aberrations, scoring was discontinued.

#### Association with outcome

The relationship between biomarker status and patient outcome was explored in both univariate and multivariate models. In the univariate analysis, only higher *ERCC1*-19q13 copy numbers, when analyzed as a continuous variable, were significantly associated with OS (HR: 0.54, 95% CI: 0.30-0.97, p = 0.04). Higher *ERCC1*-19q13/CEN-2 ratios and *ERCC1*-19q13 gain produced a similar trend, although non-significant (see Table 2). It should be noted that due to the low number of deletions, only the effect of *ERCC1*-19q13 gain was investigated. No relationship between biomarker status, TTR and LR were observed.

**Table 2:** Univariate survival analysis of the entire cohort

**Clinical endpoint**	**Covariate**	**HR**	**95% CI**	**p-value**
**OS**	***ERCC1*-19q13**	0.54	0.30-0.97	0.04
	***ERCC1*-19q13/CEN-2**	0.62	0.35-1.09	0.10
	***ERCC1*-19q13 gain**	0.68	0.44-1.05	0.08
**TTR**	***ERCC1*-19q13**	0.72	0.37-1.41	0.33
	***ERCC1*-19q13/CEN-2**	0.58	0.30-1.14	0.11
	***ERCC1*-19q13 gain**	0.77	0.48-1.24	0.28
**LR**^**a**^	***ERCC1*-19q13**	0.70	0.22-2.27	0.56
	***ERCC1*-19q13/CEN-2**	0.99	0.31-3.11	0.98
	***ERCC1*-19q13 gain**	0.73	0.33-1.59	0.43

^a^Rectal cancer patients only.

**Figure 4:**
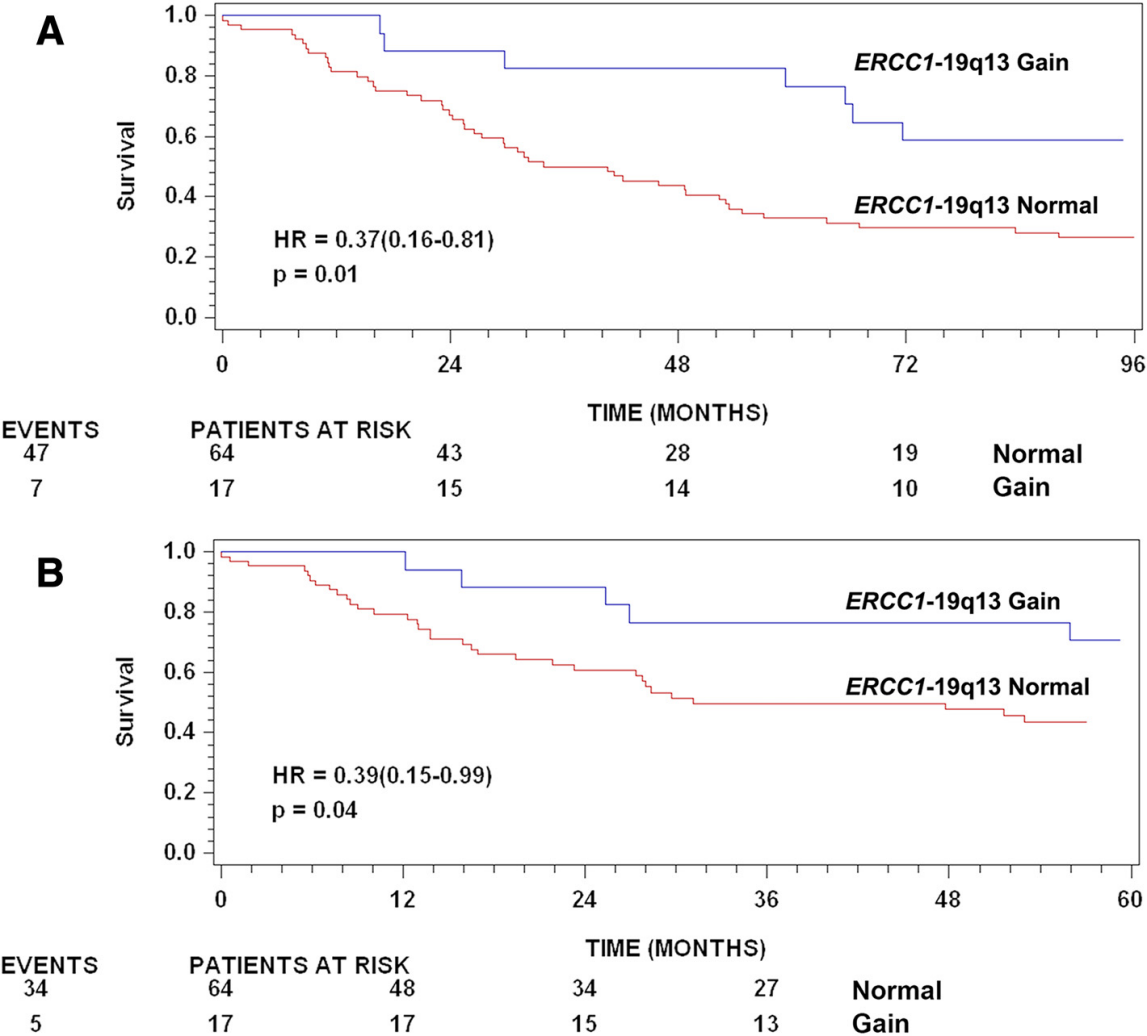
**Kaplan-Meier plots illustrating patient outcome according to *ERCC1*-19q13 status in the colon cancer subgroup. A**: Overall survival, **B**: Time to recurrence. Hazard ratios presented are Kaplan-Meier estimates of survival probabilities with corresponding 95% confidence intervals.

Multivariate analysis of age, gender and location has previously been described [17]. Tumor localization was significantly associated with both OS and TTR in the multivariate analysis where patients with rectal tumors exhibited a poorer prognosis. Similarly, higher age at time of surgery was significantly associated with poor prognosis with OS and TTR as endpoints. A test of interaction between the prognostic effect of higher *ERCC1*-19q13/CEN-2 ratios tumor localization approached significance (p = 0.07 with TTR as endpoint), and therefore the multivariate analysis (adjusting for age and gender) was performed separately for each localization. In this analysis, higher *ERCC1*-19q13 copy numbers and *ERCC1*-19q13/CEN-2 ratios were significantly associated with longer survival and TTR in patients with colon tumors, but not in patients with rectal tumors (see Table 3). Similarly, *ERCC1*-19q13 gain was significantly associated with longer survival (HR: 0.45, 95% CI: 0.20-1.00, p = 0.049) in colon tumors, whereas only a non-significant trend was observed with TTR as endpoint in the colon subgroup (HR: 0.42, 95% CI: 0.16-1.07, p = 0.07). Kaplan-Meier plots for these relationships in the colon subgroup can be viewed in Figure 4. A test of whether these effects were specific for certain colon subsites revealed no interaction between the effect of *ERCC1*-19q13 status and colon tumor subsite localization (p = 0.84). Higher gene copy numbers, ratios and *ERCC1*-19q13 gain were not associated with LR in rectal cancer patients.

**Table 3:** Multivariate survival analysis by tumor localization, adjusted for age and gender

		**Tumor Localization**					
		**Colon**			**Rectal**		
**Clinical endpoint**	**Covariate**	**HR**	**95% CI**	**p-value**	**HR**	**95% CI**	**p-value**
**OS**	***ERCC1*-19q13**	0.32	0.14-0.75	0.01	0.82	0.35-1.95	0.66
	***ERCC1*-19q13/CEN-2**	0.37	0.16-0.83	0.02	1.01	0.425-2.25	0.98
	***ERCC1*-19q13 gain**	0.45	0.20-1.00	0.049	1.01	0.59-1.75	0.97
**TTR**	***ERCC1*-19q13**	0.34	0.12-1.00	0.0499	0.87	0.36-2.15	0.77
	***ERCC1*-19q13/CEN-2**	0.24	0.09-0.70	0.01	0.87	0.36-2.14	0.77
	***ERCC1*-19q13 gain**	0.42	0.16-1.07	0.07	1.00	0.56-1.77	0.99
**LR**^**a**^	***ERCC1*-19q13**	-	-	-	0.68	0.20-2.28	0.53
	***ERCC1*-19q13/CEN-2**	-	-	-	0.91	0.28-3.04	0.88
	***ERCC1*-19q13 gain**	-	-	-	0.73	0.33-1.60	0.42

^a^Rectal cancer patients only.

### *ERCC1*-19q13 FISH scoring guidelines

To reduce observer workload, the possibility to scoring fewer nuclei to determine *ERCC1*-19q13 status was investigated. *ERCC1*-19q13 status after scoring 10, 20 and 30 nuclei was compared to the status after inclusion of all relevant nuclei. As shown in Table 4, scoring 10, 20 or 30 nuclei classified samples with moderate concordance (0.71, 0.87 and 0.89, respectively). Following the introduction of a relevant borderline interval (see material and methods) concordance was improved substantially (0.86, 0.93 and 0.99, respectively).

**Table 4:** Characteristics of updated scoring guideline

**Probe**	**Ratio cut-off**	**Borderline interval**	**Concordance**			**Mean number of scored nuclei**		
			**10 nuclei**	**20 nuclei**	**30 nuclei**	**10 nuclei**	**20 nuclei**	**30 nuclei**
***ERCC1-*** **19q13/CEN-2**	1.5	none	0.71	0.87	0.89	10	20	30
		1.35-1.65	0.86	0.93	0.99	15.3	26.4	36.5

## Discussion

A single *ERCC1*-19q13 copy number aberration was observed in the CRC cell line panel. In HT29, a total of four *ERCC1*-19q13 signals were detected in this near-triploid cell line. The finding is somewhat similar to the two different karyotypic descriptions available in the NCBI and NCI’s SKY/M-FISH & CGH database. Both descriptions list HT29 as harboring a normal copy of chromosome 19 and differ with regards to whether the cell line additionally harbors either a 19q isochromosome and a derivative fusion chromosome with material from 17q fused at 19qter [20], or a derivative 19q isochromosome with a duplication of 19q13.1-13.4 and an unbalanced translocation of 19q12-qter to chromosome 17 [21]. In the current study it appears that the additional signals from *ERCC1*-19q13 may be attributed to the formation of a 19q isochromosome fused with an additional (at least partial) duplicated 19q chromosome fragment. Taken together, these different karyotypes indicate that this region appears to be unstable in HT29. No *ERCC4* aberrations were detected.

Analysis of the 152 tumor specimens with *ERCC1*-19q13/CEN-2 revealed the presence of both gene deletions and gene gains. To determine whether the two tumor specimens with an ‘*ERCC1*-19q13 Deletion’ status were correctly classified, mean gene and reference signals were compared to those previously acquired from unaffected colon mucosa [16,17]. In the first sample, CEN-2 signal counts were in the diploid range, whereas gene signals were in the haploid range, indicative of gene deletion. In the second sample, *ERCC1*-19q13 counts were in the diploid range, while those of CEN-2 were in the triploid range, suggesting that the low ratio observed for this specimen may be attributed to either loss of *ERCC1*-19q13 in a triploid tumor, or chromosome 2 aneusomy. Taken together, these findings suggest that loss of *ERCC1*-19q13 occurs infrequently in stage III CRC.

Copy number alterations of the *ERCC1* locus are not widely reported. In ovarian cancer, *ERCC1* appears not to frequently undergo copy number aberrations [12], whereas it is a more common phenomenon in glioma [13] (note: blotting-based method were applied in both studies). In non-small cell lung cancer, *ERCC1* gene copy number increases were detected by FISH in 25.5% of samples a finding comparable to the 27.0% reported in the present study, although the FISH probe design and scoring differed substantially [14]. In this study, Vanhecke and colleagues applied an *ERCC1* gene probe in combination with a reference probe directed at 19p13 and followed *EGFR* consensus scoring guidelines to classify samples as either normal, high polysomy or gene amplified [14]. It should be noted that the use of a reference probe directed at 19p does not allow the detection of *ERCC1* gene copy increases which occur independently of the rest of 19q, i.e. gene amplification-driven copy number increases, which presents a flaw in the design of their probe.

Gain of 19q has previously been reported in colorectal cancer [22,23], indicating that a reference probe located on 19q would be ideal in differentiating between arm-level 19q gains and those involving a smaller chromosomal fragment, such as an amplicon. In the present study, the use of an *ERCC1*-19q13/CEN-2 ratio cut-off of 1.5 in combination with CEN-2, allows detection of gene copy number increases of 50% or more relative to tumoral ploidy levels. Therefore, the assay does not distinguish between gains due to large chromosomal events, such as chromosome 19 polysomy or 19q isochromosome formation, and those due to gene amplification, which involve an amplicon. To determine whether focal amplification occurs in CRC, GISTIC analysis (Genomic Identification of Significant Targets in Cancer, [24]) of CRC samples (128 tumor specimens and 33 cell lines) available in the public tumorscape database (broadinstitute.org/tumorscape) was performed [25]. The results of this analysis suggest that *ERCC1* does not undergo focal amplification.

As previously mentioned, the *ERCC1*-19q13 FISH probe covers several other genes, including *ERCC2*, *FOSB*, *PPP1R13L, MARK4* and *GPR4*. ERCC2 (also known as XPD) is a 5′ → 3′ helicase involved in unwinding the double stranded DNA structure around the DNA lesion in NER prior to ERCC1-ERCC4 incision [4]. Copy number alterations of this gene have previously been reported, but only in the form of infrequent gene loss [12,13]. These findings are in line with the deletion frequency observed in the present study. It should be noted that FOSB, PPP1R13L and GPR4 appear to play a role in oncogenesis [26-28], but do not appear to have been investigated in relation to gene copy number alterations. Interestingly, *MARK4* appears to undergo gene amplification in glioblastoma cell lines, resulting in overexpression the MARK4L isoform and increased proliferate capacity [29]. Due to the nature of the *ERCC1*-19q13/CEN-2 FISH probe, it is unknown to what extent other 19q genes are gained in specimens harboring an *ERCC1*-19q13 gain. Gain of 19q would result in increased copy number of several well known genes with involvement in cancer, such as *BAX*[30], *CEACAM1*[31,32], *AKT2*[33] and *BCL2L12*[34].

Higher *ERCC1*-19q13 copy numbers were significantly associated with longer survival in the univariate analysis, whereas higher *ERCC1*-19q13/CEN-2 ratios and *ERCC1*-19q13 gain produced non-significant trends (see Table 2). No relationships were observed for TTR and LR as clinical endpoints in the univariate analysis.

In the multivariate analysis, tumor localization was significantly associated with OS and TTR, where rectal cancers exhibited a poorer prognosis [17]. This finding may be attributed to the conventional surgical techniques at the time of specimen collection, which was performed before the introduction of total mesorectal excision (TME) in Denmark. TME has since its implementation as a standard operative procedure significantly improved overall 5-year survival, with the greatest improvement observed for stage III patients [35]. Therefore, the clinical outcome of patients in the rectal cancer subgroup is likely to differ from those receiving surgery today. It could be of future interest to investigate the relationship between *ERCC1*-19q13 gain and stage III rectal cancer patient prognosis.

Multivariate analysis, performed adjusting for age and gender, revealed significant relationships between higher *ERCC1*-19q13 copy numbers and *ERCC1*-19q13/CEN-2 ratios and longer survival and TTR in patients with colon tumors, but not rectal tumors (see Table 3). Similarly, *ERCC1*-19q13 gain was significantly associated with longer survival and exhibited a non-significant trend towards longer TTR in the colon subgroup. This non-significant finding may be attributed to the low number colon tumor specimens (17 of 81 specimens) harboring an *ERCC1*-19q13 gain. Taken together, these findings suggest that *ERCC1*-19q13 copy number increases occur in both colon and rectal tumors, but are only related to better prognosis in patients with colon tumors. Colon and rectal tumors are widely studied as a single entity and a landmark genome-scale analysis has revealed striking similarities between tumors from either localization, with the exception of tumors located in the right/ascending colon, which frequently exhibit microsatellite instability (MSI) [22]. While tumors may exhibit similar genomic profiles, the prognostic impact of a given genetic alteration may differ according to tumor localization. A study of *TP53* mutations, specifically denaturing mutations, revealed significant prognostic impacts in some tumor localizations (distal colon), but not others (proximal colon and rectum) [36], a finding with similarities to that of the present study.

After analysis of the first 50 tumor specimens, *ERCC4*/CEN-16 hybridization was terminated due to a lack of aberrations, a finding similar to what was observed in the cell line metaphase panel and also supported by GISTIC analysis in the tumorscape database. In ovarian cancer, *ERCC4* mRNA expression has previously been shown to be tightly correlated with *ERCC1* mRNA expression, indicating that the mechanisms regulating the expression of these genes are linked [37]. We therefore suggest that future explorative studies of *ERCC1* copy number alterations in other cancer types also investigate *ERCC4* copy numbers, as these may potentially play a role in *ERCC1* expression.

Observer workload can be reduced substantially by requiring fewer nuclei to be scored when determining *ERCC1*-19q13 status. Wolff and colleagues [38] suggest a minimum concordance of 0.95 as a requirement for the validation of a *HER2* assay when compared to a validated method, a guideline which was adopted for the current study. Scoring an initial 30 nuclei containing both gene and centromere signals produced concordance of 0.89, when compared to scoring all relevant nuclei. By introducing a borderline interval between 1.35 and 1.65, where an additional 30 nuclei must be scored, concordance increased to 0.99, surpassing the guidelines set forth by Wolff *et al.* In the specimens scored in the present study, these updated guidelines would have reduced the amount of nuclei scored by 30.6%. We therefore suggest that future investigations with *ERCC1*-19q13/CEN-2 in CRC score nuclei based upon the aforementioned updated guidelines.

## Conclusions

In conclusion, *ERCC1*-19q13 gain occurs in a significant fraction of CRC tumors, whereas deletion of this locus occurs infrequently and was only observed in colon tumors. In the multivariate analysis, higher *ERCC1*-19q13 copy numbers were significantly associated with longer survival and TTR, but only in patients with colon tumors. Similar results were observed for the *ERCC1*-19q13/CEN-2 ratio, supporting the use of CEN-2 as a marker for chromosomal ploidy levels. It should be noted that while no relationship between *ERCC1*-19q13 status and patient prognosis was observed in patients with rectal tumors, this does not rule out the possibility that *ERCC1-*19q13 status could potentially be linked to oxaliplatin sensitivity in both colon and rectal tumors. Future plans include testing the *ERCC1*-19q13/CEN-2 probe combination in an oxaliplatin-treated patient cohort to investigate whether *ERCC1*-19q13 status is related to response to oxaliplatin. Furthermore, the exact mechanisms behind generating higher ERCC1/ERCC4 expression levels have yet to be fully elucidated. The downstream effects of *ERCC1*-19q13 gain or deletion have not been investigated in the current study; however these copy number alterations may provide the basis for increased ERCC1/ERCC4 protein expression in a gene dosage-dependent manner, a hypothesis which requires testing with validated ERCC1 and ERCC4 antibodies.

## Competing interests

David Hersi Smith and Sven Müller are employed at Dako A/S. Ib Jarle Christensen is an external consultant for Dako A/S. The remaining authors have no disclosures or conflicts of interest.

## Authors’ contributions

DHS – collection and analysis of data, preparation of manuscript. IJC and BM – statistical analysis of data and preparation of manuscript. NFJ – prepation of metaphase specimens and manuscript. SVM – FISH probe design and preparation of manuscript. HJN - provided patient samples and preparation of manuscript. NB – preparation of manuscript and experimental design. KVN – preparation of manuscript and experimental design. All authors read and approved the final manuscript.

## Pre-publication history

The pre-publication history for this paper can be accessed here:

http://www.biomedcentral.com/1471-2407/13/489/prepub
